# Transcriptional signatures of salinity tolerance in Egyptian wheat: unveiling WRKY-mediated defense mechanisms

**DOI:** 10.1186/s12870-026-08483-0

**Published:** 2026-03-23

**Authors:** Amira A. Ibrahim, Marwa A. Fakhr, Samah Ramadan, Mostafa M. Basuoni, Mohamed Abdel-Haleem

**Affiliations:** 1 Botany and Microbiology Department, Faculty of Science, Arish University, Al-Arish, 45511 Egypt; 2 Botany Department, Faculty of Science, Fayoum University, Fayoum, 63514 Egypt; 3 Botany Department, Faculty of Science, Mansoura University, Mansoura, Egypt; 4 Botany and Microbiology Department, Faculty of Science (Boys), Al- Azhar University, Cairo, 11884 Egypt; 5 Botany and Microbiology Department, Faculty of Science, Zagazig University, Zagazig, 44519 Egypt

**Keywords:** *Triticum aestivum* L., Stress tolerance, WRKY factors, Molecular characterization, ROS.

## Abstract

**Background:**

Salinity stress severely constrains wheat productivity by impairing growth, photosynthetic efficiency, and cellular redox homeostasis. Understanding the physiological and transcriptional mechanisms underlying salinity tolerance is essential for developing resilient wheat cultivars.

**Results:**

In this study, four Egyptian wheat (*Triticum aestivum* L.) genotypes (Giza 171; Sakha 95; Gemmiza 11, and Misr 3) were evaluated under 0 (control), 6.25, and 12.5 dS m⁻¹ NaCl to elucidate genotype-specific physiological, biochemical, and molecular responses to salinity stress. Increasing salinity significantly reduced germination percentage, biomass accumulation, and leaf chlorophyll content, with more pronounced inhibition at 12.5 dS m⁻¹, particularly in salt-sensitive genotypes. Salinity stress markedly increased oxidative damage, as indicated by elevated hydrogen peroxide (H₂O₂) and malondialdehyde (MDA) levels, and increased electrolyte leakage. In contrast, salt-tolerant genotypes (Sakha 95 and Giza 171) exhibited enhanced antioxidative capacity, characterized by increased activities of superoxide dismutase, catalase, ascorbate peroxidase, glutathione reductase, and polyphenol oxidase, as well as higher accumulation of phenolic and flavonoid compounds. Transcriptional analysis revealed significant salinity-induced upregulation of WRKY transcription factors (WRKY1, WRKY20, WRKY33, and WRKY53), as well as TaSOS1 and LEA-1, with stronger induction in tolerant genotypes, indicating coordinated regulation of ion homeostasis, oxidative stress defense, and cellular protection mechanisms.

**Conclusions:**

Our findings demonstrate that salinity tolerance in wheat is governed by an integrated network linking physiological performance, antioxidant defense, and transcriptional reprogramming of key stress-responsive genes. Sakha 95 and Giza 171 emerge as promising genetic resources for breeding programs to enhance salinity tolerance in wheat.

**Supplementary Information:**

The online version contains supplementary material available at 10.1186/s12870-026-08483-0.

## Introduction

Abiotic stresses, particularly drought and heat, impose significant constraints on global crop productivity by disrupting normal plant development and impeding the completion of crop life cycles, especially amid rapidly evolving climatic conditions [1, 2]. In recent decades, the frequency and intensity of drought and heat events during various growth phases have increased markedly. Plants utilize various morpho-physiological and biochemical pathways to mitigate these stresses [3, 4]. Two primary strategies employed by plants to cope with reduced water availability and elevated temperatures are avoidance and tolerance mechanisms [5, 6]. Drought stress adversely impacts critical physiological and biochemical processes, leading to hormonal imbalances characterized by heightened ethylene production and diminished photosynthetic efficiency [7–9]. The extent of these adverse effects is contingent upon the intensity, duration, and specific growth stage during which the stress occurs [10].

While seed yield is commonly viewed as the principal criterion in germplasm evaluation, effective breeding strategies require identifying key morpho-physiological and biochemical traits that enhance heat and drought tolerance [11, 12]. The aforementioned stresses adversely influence plant growth by reducing leaf size and number, lowering chlorophyll and osmolyte levels, decreasing turgor pressure, and impairing the photosynthetic apparatus, ultimately resulting in diminished photosynthetic rates [13–16]. Drought and heat stress in oilseed crops lead to significant reductions in both the quantity and quality of oil [17–20].

Wheat (*Triticum aestivum* L.) serves as a crucial global staple crop, with demand projected to increase by 60% by 2050 [21]. Wheat production is substantially affected by various abiotic stresses, particularly salinity, drought, and heat [22]. Periodic drought episodes markedly reduce wheat productivity in arid and semi-arid regions [23]. Thus, the development of drought-adapted and stress-resilient wheat cultivars is vital for sustaining agricultural growth in these environments [24]. Salinity constitutes a significant and pervasive abiotic stress that severely affects plant performance, particularly in arid conditions [25–27]. Approximately 30% of irrigated land, which contributes nearly one-third of global food production, is affected by salinization [28, 29]. Furthermore, about 35% of Egypt’s agricultural land is affected by salinity [30]. Climate change, characterized by reduced precipitation and heightened temperatures, exacerbates soil salinization through limited leaching and increased evapotranspiration [31].

Salinity stress detrimentally impacts plant growth through osmotic stress and ion toxicity, arising from the accumulation of excessive Na⁺ and Cl⁻ in leaves and the root zone [32, 33]. The inhibition of essential ion uptake, including K⁺, Mg²⁺, and Ca²⁺, disrupts nutrient balance, leading to cellular ionic imbalances [34, 35]. Furthermore, salinity leads to the overproduction of reactive oxygen species (ROS), such as O₂⁻, OH⁻, H₂O₂, and ¹O₂, which can damage nucleic acids, pigments, membranes, and proteins, and impair CO₂ assimilation and photosynthetic efficiency [36–40]. Salinity markedly influences plant growth, development, and overall yield [41–44].

Plants mitigate ROS-induced cellular damage through a robust antioxidant defense system, which comprises both enzymatic components, specifically superoxide dismutase (SOD), catalase (CAT), and peroxidase (POD) and non-enzymatic osmolytes, including soluble sugars, proline, and ascorbic acid [45]. Late embryogenesis-abundant (LEA) proteins contribute to stress tolerance by protecting cellular structures under conditions of drought, low temperature, salinity, or hormonal signals [46, 47]. The Salt Overly Sensitive (SOS) signaling pathway is crucial for maintaining Na⁺ and K⁺ homeostasis during salinity stress. WRKY transcription factors (TFs) represent one of the most prominent families of plant-specific TFs and serve as essential regulators of plant responses to both biotic and abiotic stresses [48, 49].

Transcription factors (TFs) contain one or two highly conserved WRKY domains, each approximately 60 amino acids in length. They are classified into three main groups based on the number of domains and the type of zinc-finger motif [50]. These factors primarily interact with W-box cis-regulatory elements (TTGACT/C) found in the promoters of target genes [51], although alternative binding sequences have also been identified [52, 53]. Genome-wide identification of WRKY genes has been conducted across multiple plant species, highlighting their critical roles in regulating stress-responsive pathways [54–58]. Although several transcriptomic and candidate-gene studies have investigated salinity responses in wheat, the regulatory networks linking transcription factor activity to downstream stress-responsive pathways remain incompletely elucidated. The role of WRKY transcription factors in genotype-specific transcriptional reprogramming under salinity stress, as well as their relationship with genes involved in ion homeostasis and oxidative stress control, remains inadequately characterized [59, 60]. We posited that salt-tolerant wheat genotypes exhibit temporally regulated, synchronized activation of WRKY genes, along with essential downstream targets such as TaSOS1 and LEA-1, establishing a regulatory framework that distinguishes tolerant from sensitive genotypes. To evaluate this notion, we conducted a comprehensive gene expression investigation across diverse wheat genotypes subjected to escalating salinity levels at specific developmental phases [60, 61].

This study seeks to elucidate the molecular foundation of salinity tolerance in bread wheat (*Triticum aestivum* L.) by analyzing genotype-specific transcriptional responses of critical salt-responsive genes at varying salinity levels (6.25 and 12.5 dS m⁻¹ NaCl). The objectives are to: (i) analyze the expression dynamics of WRKY transcription factors and other stress-responsive genes related to ion homeostasis, oxidative stress regulation, and cellular protection across wheat genotypes with differing genetic backgrounds; (ii) categorize genotypes based on salinity tolerance through integrated gene expression profiles and seedling performance; and (iii) clarify the transcriptional–physiological relationships that drive varied salinity responses, thereby identifying candidate genes and regulatory patterns pertinent to wheat enhancement under saline conditions.

## Materials and methods

### Plant material

Four wheat (*Triticum aestivum* L.) cultivars—Giza (Gi 171), Sakha (Sk 95), Gemmiza (Gm 11), and MISR (Mi 3)—were used in this study for all experimental analyses.

## Experimental setup and treatments

The experiments were conducted at Mansoura University, Egypt. Pot experiments took place in a greenhouse from September 2025 to October 25 2025. During this period, the relative humidity ranged from 60% to 70% both day and night, while temperatures fluctuated between 18 °C and 25 °C during the day and from 9 °C to 19 °C at night as presented in Table S1. The plastic pots, each measuring 10 cm in diameter and 12 cm in depth, were filled with a 4:1 mixture of clay soil and compost. Prior to sowing, nutrients were supplemented with 0.2 g of ammonium nitrate (NH₄NO₃) and 0.2 g of potassium dihydrogen phosphate (KH₂PO₄) per pot to provide nitrogen, phosphorus, and potassium. In laboratory germination studies, 15 sterilized seeds from each cultivar were placed on Whatman No. 1 filter paper within Petri dishes. Salinity treatments were administered by augmenting irrigation water with NaCl to attain final electrical conductivity (EC) levels of 6.25 dS m^−1^ (approximately 4000 ppm) and 12.5 dS m⁻¹ (approximately 8000 ppm).

In contrast, control plants received irrigation with tap water (about 0.6 dS m⁻¹). To prevent osmotic shock, NaCl was administered gradually over 3 days until the desired electrical conductivity was achieved. Subsequently, salinity treatments were maintained for three weeks.

Plants were cultivated in plastic pots containing a homogenized soil mixture, with irrigation meticulously regulated to sustain soil moisture near field capacity while reducing drainage and leaching losses. Water quantities were standardized across treatments, and pots were irrigated to maintain consistent water availability based on weight loss. To confirm that the desired salinity levels were consistently maintained, soil electrical conductivity was periodically assessed throughout the trial using saturated soil paste extracts from representative pots for each treatment. The measurements verified that soil EC values consistently aligned with the target levels during the experiment, signifying stable salinity exposure. Pots were arranged in a fully randomized design and frequently relocated to mitigate positional effects within the greenhouse. 

Germination percentage (GP%) was determined after a seven-day incubation period. For the pot experiment, twenty surface-sterilized seeds were sown in each pot. During the first week after sowing (0–7 days after sowing; DAS), all pots were irrigated daily with quarter-strength Hoagland nutrient solution to ensure uniform seedling establishment. Salinity stress treatments were subsequently imposed by supplementing the irrigation solution with NaCl to achieve electrical conductivity levels of 6.25 and 12.5 dS m⁻¹. Salt stress was applied continuously from 7 DAS onward. Physiological, biochemical, and molecular analyses were performed at 17 and 24 DAS, corresponding to 10 and 17 days of salinity exposure, respectively. Each treatment consisted of five independent replicates, with each replicate represented by a single pot.

### Seedling growth parameters

Seedlings were harvested at 10 and 20 days after salt stress application for growth analysis. Three plants were carefully removed from each pot, thoroughly washed to eliminate soil, and measured for the following parameters: plant height (cm), hoot fresh weight (SFW) and root fresh weight (RFW), shoot dry weight (SDW) and root dry weight (RDW) after oven-drying at 80 °C for 48 h.

### Photosynthetic pigments

The chlorophyll content in the uppermost fully expanded leaves was assessed through spectrophotometry. Leaf samples (0.2 g) were incubated in 10 mL of 80% aqueous acetone, followed by centrifugation. The absorbance of the supernatant was measured at 645 and 663 nm [62].

### Oxidative stress markers

#### Hydrogen Peroxide (H₂O₂)

Leaf samples were extracted using trichloroacetic acid and subsequently centrifuged. The supernatant was treated with phosphate buffer and potassium iodide, and absorbance was recorded at 390 nm to quantify H₂O₂ concentration [63].

#### Malondialdehyde (MDA)

The MDA content was assessed by homogenizing leaf samples in ethanol, followed by a reaction with thiobarbituric acid (TBA) in trichloroacetic acid (TCA), boiling, cooling, and centrifugation. Absorbance was recorded at 532 nm and adjusted at 600 nm [64].

#### Electrolyte Leakage (EL)

Leaf discs were incubated in deionized water, and electrical conductivity (EC) was assessed at three intervals: initial (EC1), post-incubation at 55 °C (EC2), and following boiling at 100 °C (EC3). The percentage of electrolyte leakage was calculated as follows [65]:

Electrolyte leakage (%) = {(EC2- EC1) /EC3} ×100.

### Soluble protein content

Quantification of soluble proteins was performed utilizing the Bradford method. Leaf extracts were treated with Folin phenol reagent, and the absorbance was recorded at 700 nm, utilizing bovine serum albumin as a standard [66].

### Antioxidant enzyme activities

One gram of fresh leaf tissue was homogenized in chilled 50 mM phosphate buffer (pH 7.0) with 1% polyvinylpyrrolidone and 1 mM EDTA. Homogenates underwent centrifugation at 15,000 × g for 20 min at 4 °C. The supernatant was utilized for enzyme assays. The activity of Superoxide Dismutase (SOD, EC 1.15.1.1) was assessed by observing the inhibition of nitroblue tetrazolium (NBT) photoreduction at 560 nm [67]. Catalase (CAT, EC 1.11.1.6) activity was assessed by monitoring the decrease in absorbance at 240 nm due to the decomposition of H₂O₂ [68]. The activity of Ascorbate Peroxidase (APX; EC 1.11.1.11) was assessed by observing the oxidation of ascorbate at a wavelength of 290 nm [69]. Glutathione Reductase (GR, EC 1.6.4.2) activity was measured spectrophotometrically according to established protocols [70]. Peroxidase activity (POX, EC 1.11.1.7) was determined using the Lück [71] method with p–phenylenediamine as the substrate, and absorbance was measured at 485 nm.

### Total phenolic and flavonoid content

The measurement of total phenolic content (TPC) [72] and total flavonoid content (TFC) was conducted through colorimetric assays [73].

### Gene expression analysis

The Plant RNA Kit from Sigma‒Aldrich was utilized for this procedure. After purification, the RNA was analyzed on a 1% agarose gel and quantified by spectrophotometry. Each sample comprised the following components in the reaction mixture: 10 µg of total RNA, of which 5 µg was subjected to reverse transcription. The composition comprised 10 pmL/µL oligo dT primer, 2.5 µL of 5X buffer, 2.5 µL of MgCl_2_, 2.5 µL of 2.5 mM dNTPs, 4 µL of oligo dT, 0.2 µLof 5 units/µL reverse transcriptase from Promega, Germany, and 2.5 µL of RNA. The thermal cycler for PCR was set for RT-PCR amplification at 42 °C for 1 h and 72 °C for 20 min. The Rotor-Gene 6000 system, an advanced German technology, was used for real-time PCR analysis. The analysis used 1 µL of diluted cDNA in triplicate to ensure the precision and reliability of the results. The primers utilized for qRT‒PCR are presented in Table 1. A SYBR^®^ Green-based method was employed for gene expression analysis, utilizing primers for four distinct genes. The total reaction volume was 20 µL. A minimal quantity of template, a designated volume of SYBR Green Master Mix, reverse and forward primers, and sterile distilled water were meticulously included in the mixture. The PCR conditions employed were as follows: the temperature was elevated to 95 °C for 15 min, subsequently lowered to 60 °C for 30 s; this cycle was reiterated 40 times. The CT values of the target gene were subtracted from those of the β-actin gene to obtain the ΔCT value. Livak and Schmittgen [74] used the 2^^−ΔΔCt^ method to determine relative gene expression.

**Table 1:** Oligonucleaotide primers used in qRT-PCR analysis

Gene Name		Sequence
TaWRKY1	F	5ʹAGTTCCCTGCTATTCCATCTAAG-3ʹ
	R	5ʹ- TTCTCGCTCGCCATCTCC-3ʹ
TaWRKY20	F	5ʹ- CACCACCACCACCACCTC-3ʹ
	R	5ʹ- AGCAGCGACGACGACATC-3ʹ
TaWRKY33	F	5ʹ- CCACCTCCTTCACTTCCATT − 3ʹ
	R	5ʹ- GATGGAAAACTCCCAGTCGT − 3ʹ
TaWRKY53	F	5ʹ- CACATACCGAGGCTCCCATAA − 3ʹ
	R	5ʹ- CCTGTTGGATAAACGGCTTGG − 3ʹ
*TaSOS1*	F	5´- AGCGTGTCGTATCCAAA − 3’
	R	5´- GTCGTCATCTTCTCCTACC − 3’
Late embryogenesis abundant proteins (LEA-1)	F	5´- CAGCGAAGTTTGGATGGAATG-3’
	R	5´- ACCTGTCGCCAATCAGAAGAT-3’
β-Actin	F	5´-GTGCCCATTTACGAAGGATA- 3´
	R	5´-GAAGACTCCATGCCGATCAT- 3´

### Statistical analysis

All experimental results were subjected to statistical analysis and are presented as means ± SD. The Shapiro-Wilk normality test was used to assess normality at the 0.05 significance level. The statistical significance of treatment differences was assessed using a one-way ANOVA followed by Tukey’s multiple comparisons test, with a significance threshold of *P* < 0.05. Data were analyzed with GraphPad Prism 9 (GraphPad Software, Inc., San Diego, CA, USA). The intercorrelations (scatter coefficient, heatmap correlations, principal component analysis (PCA)) among various morphological and biochemical parameters of the examined wheat genotypes under varying salinity stress concentrations were analyzed using the R Studio interface and R software, resulting in the creation of a heatmap matrix for multivariate analysis (R Studio Team [75]; R Core Team [76]).

## Results

### Morphological responses of *Triticum aestivum* seedlings to salinity stress

**Fig. 1:**
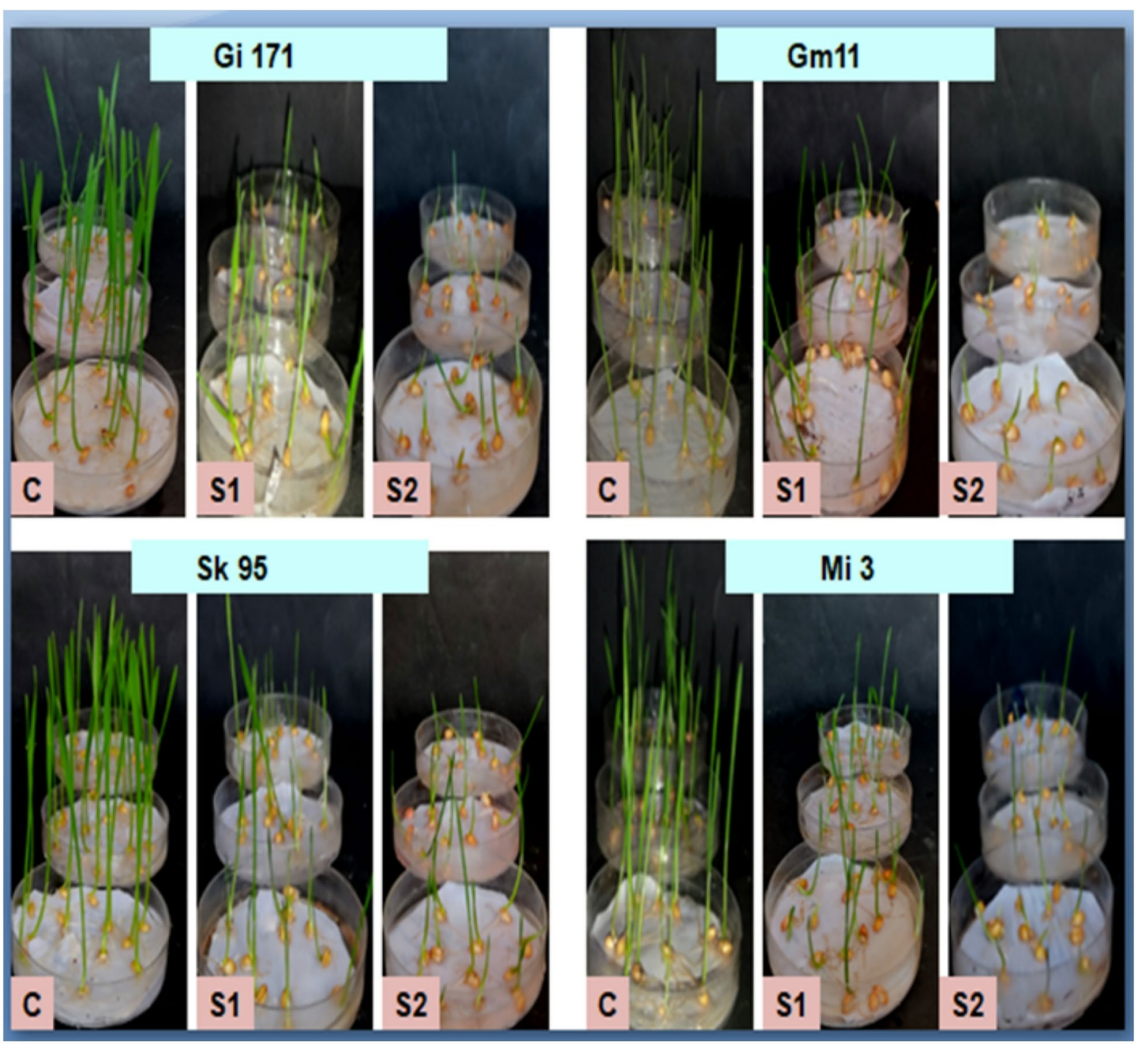
Photographs illustrated morphology of the examined four wheat genotypes grown in Laboratory condition using water (control) and under salinity levels (6.25 dSm^− 1^, and 12.5 dSm^− 1^ NaCl) at 17 days old seedling

**Fig. 2:**
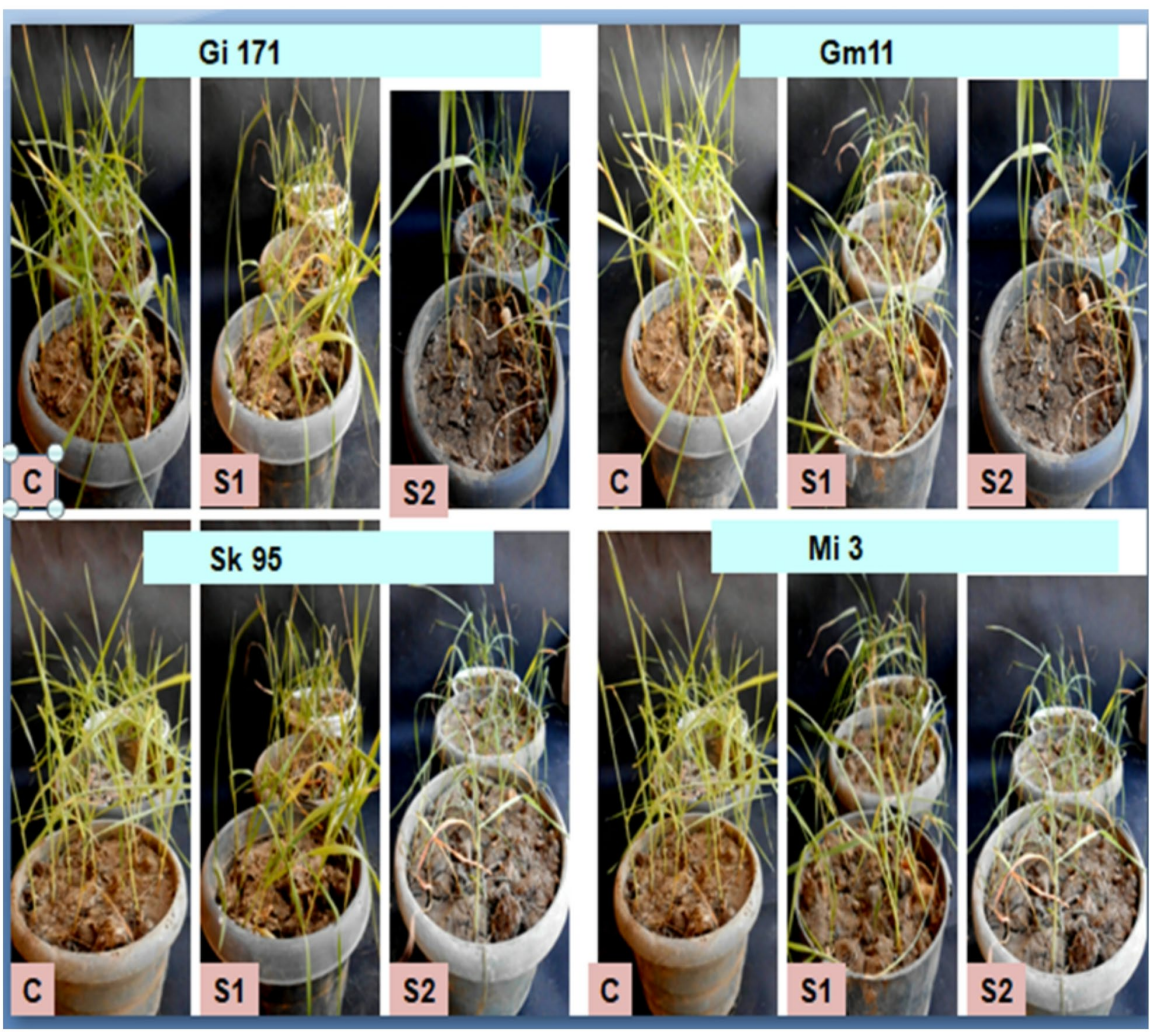
Photographs illustrated morphology of the examined four wheat genotypes grown in Laboratory condition using water (control) and under salinity levels (6.25 dSm^-1^, and 12.5 dSm^-1^ NaCl) at 24 days old seedling

The influence of salinity on germination and initial seedling development was evaluated across four *Triticum aestivum* cultivars: Giza 171, Gemmiza 11, Sakha 95, and Misr 3. The experimental results demonstrated varying responses among genotypes to mild (S1, 6.25 dSm⁻¹ NaCl) and severe (S2, 12.5 dSm⁻¹ NaCl) salinity stress at 17 and 24 days after sowing (DAS), as shown in Table S1 and Figures (1 & 2).

### Effects of salinity on germination and seedling growth

#### Seed germination

Salinity had a moderate inhibitory effect on the final germination percentage (GP%). Under controlled conditions, all cultivars achieved 100% germination. Mild salinity stress (S1) resulted in a minor decrease in GP%, with the tolerant genotypes Sakha 95 and Giza 171 sustaining GP% values of 97–98% and 95–96%, respectively. In contrast, the moderately sensitive cultivars Misr 3 and Gemmiza 11 exhibited more significant reductions, reaching 89–91% and 86–87%, respectively. At elevated salinity levels (S2), germination rates were notably diminished, especially in sensitive cultivars, with Misr 3 and Gemmiza 11 achieving germination percentages of 86–87% and 81–82%, respectively. In contrast, tolerant genotypes maintained higher germination percentages, with Sakha 95 recording 94–95% and Giza 171 showing 91–93% (Table S2). The findings suggest that salinity tolerance during germination is genotype-dependent.

### Plant height

**Fig. 3:**
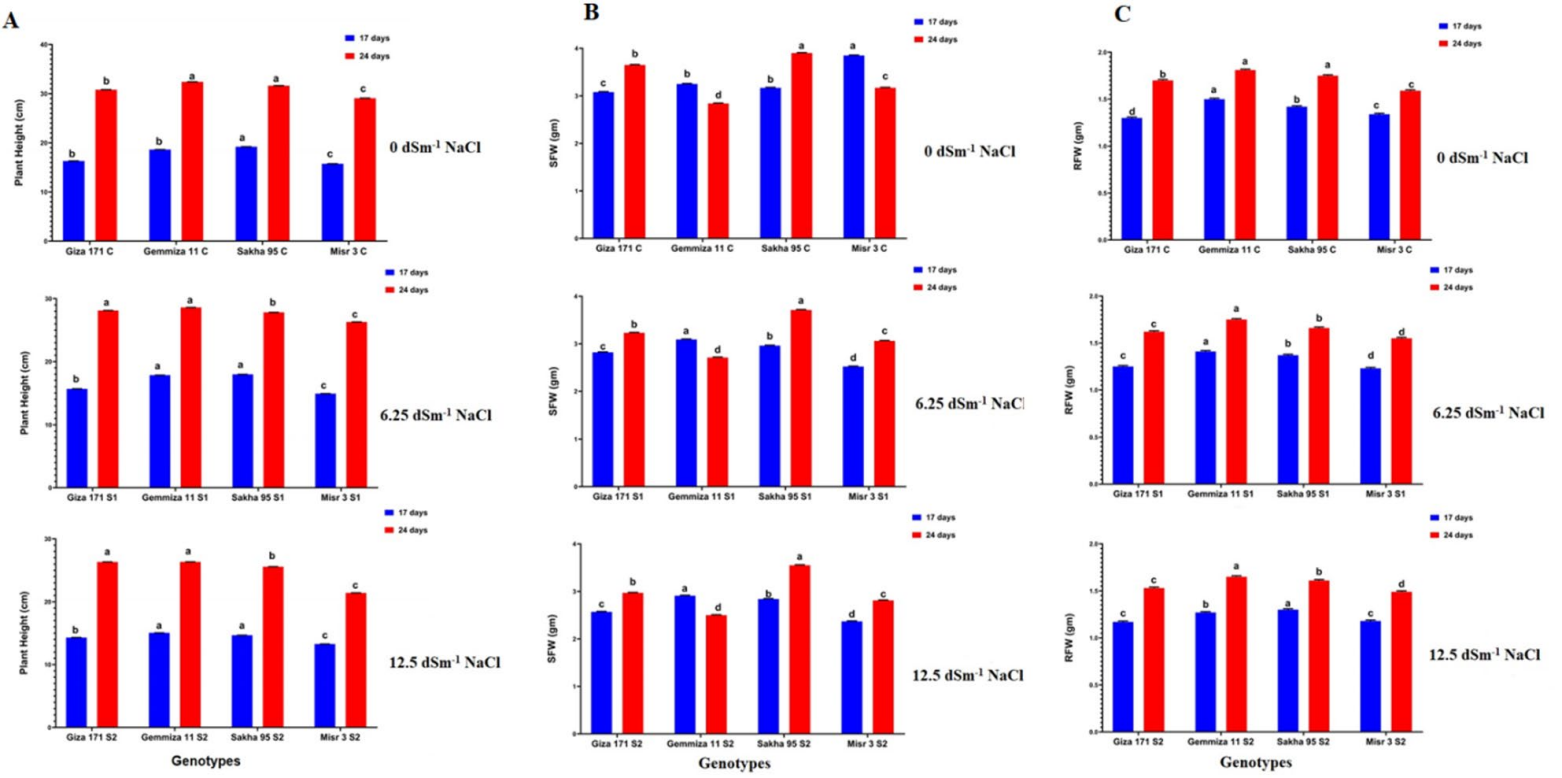
Histograms showed morphological parameter (**A**: plant height (Ph) in cm, **B**: shoot fresh weight (SFW) and **C**: root fresh weight (RFW) in g) for the studied wheat genotypes in control and salinity treatments (6.25 dSm^− 1^ and 12.5 dSm^− 1^ NaCl) at 17 and 24 days after sowing (DAS). Bars with different letters indicate significant differences between treatments, expressed as the mean of three replicates ± SDs

Salinity significantly reduced seedling height. Under severe salinity (S2), the height of Giza 171 decreased from 30.80 cm (control) to 26.33 cm at 24 days after sowing (DAS), whereas Misr 3 showed a more pronounced decline from 29.08 cm to 21.41 cm. Sakha 95 exhibited a modest reduction in height, remaining within the range of 25.56–25.58 cm, indicating its tolerance to salinity (Fig. 3A).

### Fresh and dry biomass

**Fig. 4:**
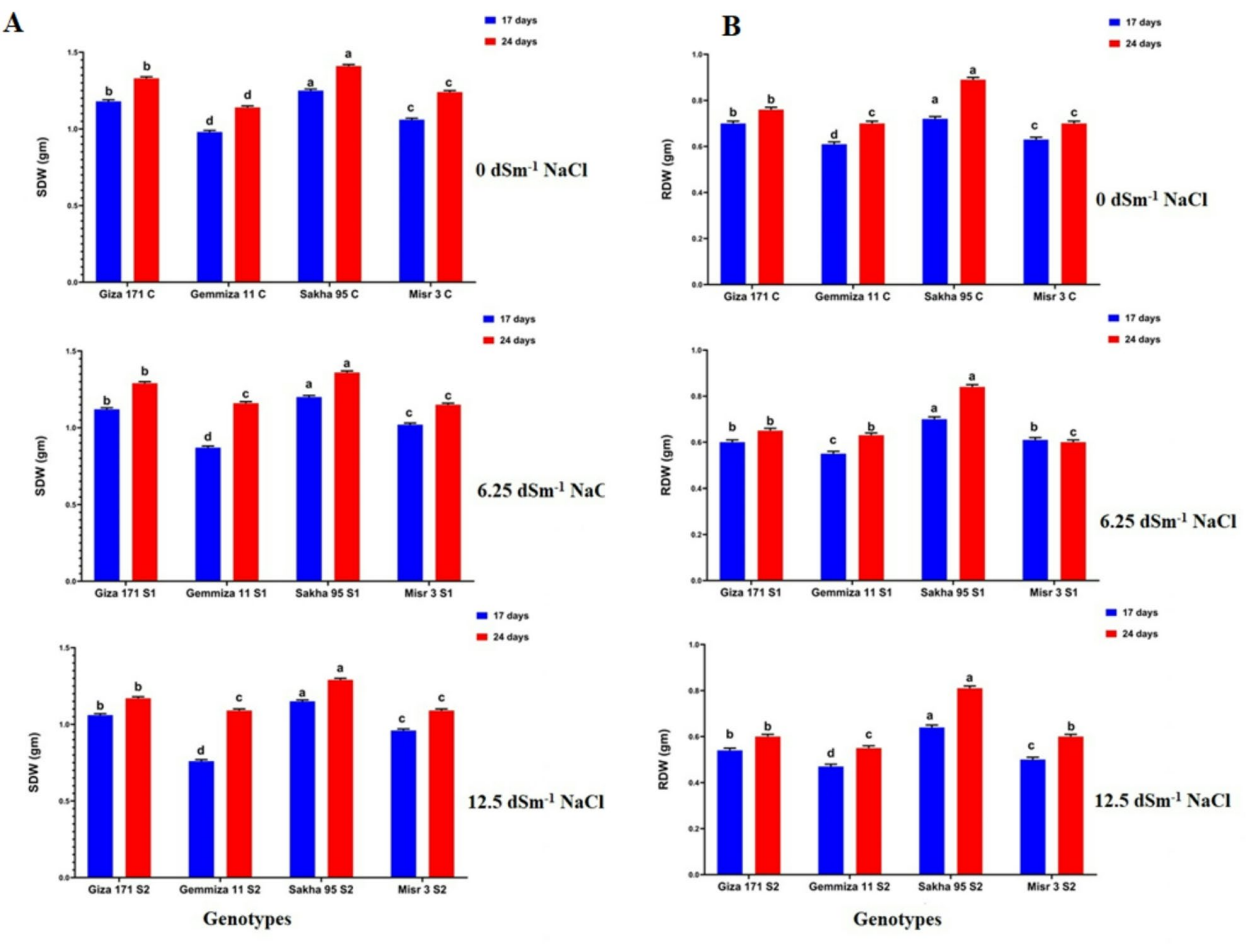
Histograms showed morphological parameter (**A**: shoot dry weight (SDW) and **B**: root dry weight (RDW) in g) for the studied wheat genotypes in control and salinity treatments (6.25 dSm^− 1^ and 12.5 dSm^− 1^ NaCl) at 17 and 24 days after sowing (DAS). Bars with different letters indicate significant differences between treatments, expressed as the mean of three replicates ± SDs

Salinity stress adversely affected both fresh and dry biomass. Tolerant genotypes, specifically Sakha 95 and Giza 171, exhibited greater shoot and root biomass under stress conditions, while sensitive cultivars, such as Misr 3 and Gemmiza 11, demonstrated significant reductions. Shoot fresh weight (SFW) showed highest decreased in Misr 3 genotype varied from 3.85 g to 2.52 g under S1 (6.25 dsm^− 1^) treatment and to 2.37 g under S2 treatment (12.5 dsm^− 1^) at 17 days after sowing (DAS) followed by Giza 171 genotype showed decreased from 3.08 g to 2.82 g and 2.57 g under S1 and S2 treatments respectively at 17 days after sowing (DAS) (Fig. 3B). The root fresh weight (RFW) recorded showed the lowest decrease in the Sakha 95 genotype, varying from 1.42 g to 1.37 g and 1.30 g under S1 and S2 treatments, respectively, at 17 days after sowing (DAS) (Fig. 3C). For example, the shoot dry weight (SDW) of Misr 3 decreased from 1.06 g in the control group to 0.96 g under S2 at 24 days after sowing (DAS) (Fig. 4B).

### Photosynthetic pigments and growth-related traits under salinity

#### Leaf Chlorophyll Content (Leaf Chl)

**Fig. 5:**
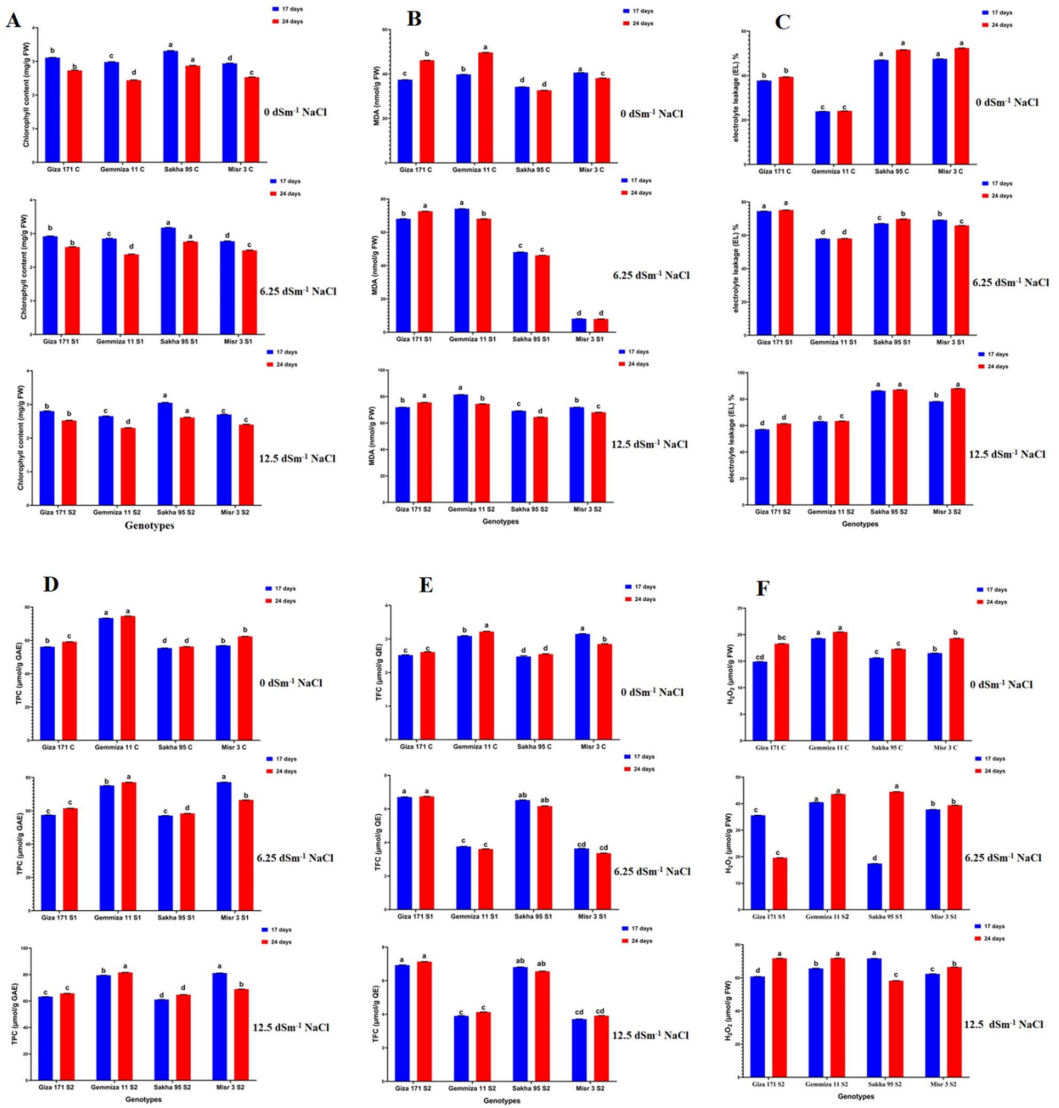
Histograms showed the effect of two salinity concentrations (6.25 and 12.5 dSm^− 1^) on **A**: chlorophyll content, **B**: MDA (Malondialdehyde), **C**: El (Electrolyte Leakage), **D**: TPC (Total Phenolic content), **E**: TFC (Total Flavonoid content) and F: H_2_O_2_ (Hydrogen Peroxide level) of *T. aestivum* genotypes grown for 17 and 24 days. Bars with different letters indicate significant differences between treatments, expressed as the mean of three replicates ± SDs

The chlorophyll content in wheat seedlings demonstrated a distinct response that was dependent on both genotype and salinity levels. Under control conditions, all genotypes exhibited relatively high chlorophyll levels, with Sakha (Sk 95) and Gemmiza (Gm 11) demonstrating the highest values at both 17 and 24 DAS, indicating strong photosynthetic potential. Sakha 95 genotypes showed the lowest decrease in total chlorophyll content, ranging from 3.31 to 3.177 mg/g FW under S1 and S2 treatments (6.25 and 12.5 dSm^-1^ NaCl) at 17 days after sowing (DAS). Also, Sakha 95 genotypes showed the lowest decrease in total chlorophyll content, ranging from 2.87 to 2.76 and 2.61 mg/g FW under S1 and S2 treatments (6.25 dSm^-1^ and 12.5 dSm^-1^ NaCl) at 24 days after sowing (DAS). Progressive salinity stress (S1 and S2) resulted in a notable reduction in chlorophyll concentration, particularly in Giza (Gi 171) and Misr (Mi 3), suggesting disruption of the photosynthetic apparatus. The reduction was significantly greater at 24 DAS, indicating cumulative stress effects over time. The findings indicate that chlorophyll degradation in response to salinity is both genotype-specific and temporally pronounced (Fig. 5A, Table S3).

### Salinity-induced oxidative stress markers

#### Malondialdehyde (MDA) content

MDA, an indicator of lipid peroxidation and oxidative stress, increased significantly under salinity stress in all genotypes. Control seedlings exhibited minimal baseline MDA levels, indicating negligible oxidative damage. Exposure to moderate (S1) and severe (S2) salinity resulted in a significant increase in MDA content. With the highest levels recorded in Misr (Mi 3) under S2 conditions, the significant increase varied from 40.61 to 71.92 nmol/g FW under S2 treatments, respectively, at 17 days after sowing, while it varied from 38.16 to 68.12 nmol/g FW at 24 days after sowing (Fig. 5B & Table S3).

#### Electrolyte Leakage (EL)

Electrolyte leakage, an indicator of membrane integrity, increased significantly under salinity stress. Control seedlings demonstrated negligible leakage, indicating the integrity of cellular membranes. Salinity exposure (S1 and S2) resulted in a significant increase in EL, with Misr (Mi 3) and Sakha (Sk 95) exhibiting the highest EL percentages under severe stress. Misr 3 genotype showed an increased ranged from 47.52 to 69.20% under S1 treatment and to 78.21% under S2 treatment at 17 DAS. While the Sakha 95 genotype showed an increase from 47.05 to 67.10 and 86.32% after S1 and S2 treatments, respectively, at 17 DAS, and varied from 51.62 to 69.81 and 87.14% after S1 and S2 treatments, respectively, at 24 DAS (Fig. 5C & Table S3). 

### Non-Enzymatic antioxidant metabolites

#### Total Phenolic Content (TPC)

Total phenolics, key secondary metabolites with antioxidative capacity, showed genotype- and stress-dependent increases. Control seedlings displayed moderate TPC levels, whereas salinity stress (S1 and S2) significantly elevated TPC accumulation, particularly in Misr (Mi 3) and Sakha (Sk 95). The increase was more pronounced at 17 DAS; Misr 3 genotypes showed a significant increase from 56.92 to 77.19 and 81.19 µmol/g GAE under S1 and S2 treatments, respectively. Also, Misr 3 showed an increase at 24 DAS, ranging from 62.41 to 66.51 µmol/g GAE under S1 and S2 treatments, respectively (Fig. 5D, Table S3).

#### Total Flavonoid Content (TFC)

Flavonoid accumulation followed a pattern similar to that of total phenolic content, indicating their role as effective antioxidants. Under salinity stress, total flavonoid content (TFC) exhibited a significant increase, particularly in Misr (Mi 3) and Giza (Gi 171) under severe (S2) conditions. The Giza 171 genotype showed a significant increase, ranging from 2.52 to 6.71 µmol/g QE under S1 and 6.93 µmol/g QE under S2 treatment, respectively, at 17 DAS; also varied from 2.61 to 6.74 µmol/g QE under S1 and 7.13 µmol/g QE under S2 treatment, respectively, at 24 DAS (Fig. 5E).

### Hydrogen Peroxide (H₂O₂) accumulation

The H₂O₂ content, a marker of reactive oxygen species (ROS), mirrored the patterns observed for MDA and EL. Under control conditions, hydrogen peroxide levels were consistently low across all genotypes. Salinity stress induced a significant increase in H₂O₂, particularly under severe (S2) conditions, with Misr (Mi 3) and Giza (Gi 171) exhibiting the highest accumulation of reactive oxygen species (ROS). Giza 171 genotype showed a significant increase, ranging from 14.91 to 35.61 µmol/g FW under S1 and 60.81 µmol/g FW under S2 treatment, respectively, at 17 DAS; also varied from 18.30 to 19.61 µmol/g FW under S1 and 71.82 µmol/g FW under S2 treatment, respectively, at 24 DAS. Misr 3 genotype showed a significant increase, ranging from 16.52 to 37.81 µmol/g FW under S1 and S2 treatment, respectively, at 17 DAS; also varied from 19.31 to 39.42 µmol/g FW under S1 and S2 treatment, respectively, at 24 DAS (Fig. 5F).

### Antioxidant Enzyme Responses to Salinity StressSuperoxide Dismutase (SOD) Activity

**Fig. 6:**
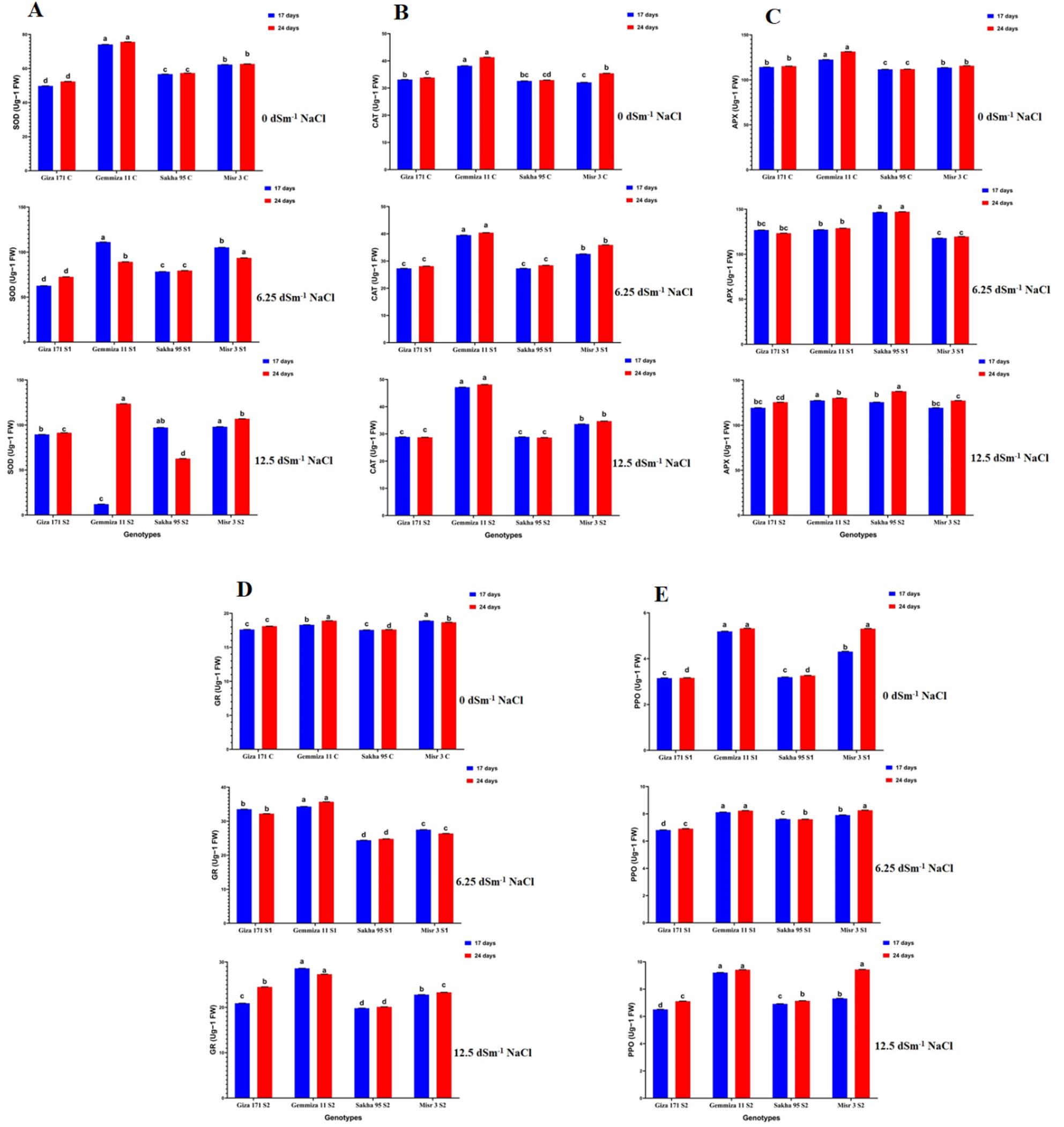
Histograms showed the effect of two salinity concentrations (6.25 and 12.5 dSm^-1^) on antioxidant enzymes activities; **A**: SOD (Superoxide Dismutase), **B**: CAT (Catalase), **C**: APX (Ascorbate Peroxidase), **D**: GR (Glutathione Reductase) and **E**: PPO (Polyphenol Oxidase) of *T. aestivum* genotypes grown for 17 and 24 days. Bars with different letters indicate significant differences between treatments, expressed as the mean of three replicates ± SDs

Salinity induced a significant increase in superoxide dismutase activity in all wheat genotypes. Under moderate stress (S1), Gemmiza (Gm 11) demonstrated the highest SOD levels at 17 DAS (111.20 ± 0.01 Ug ^− 1^ FW), indicating its enhanced ability to superoxide dismutase radicals. Misr (Mi 3) exhibited significant SOD induction in high salinity conditions (S2, 98.11 ± 0.01 Ug^ − 1^ FW at 17 DAS), while control plants sustained basal levels (~ 49–62 Ug − 1 FW). Temporal dynamics showed a gradual increase from 17 to 24 days after sowing, indicating ongoing activation of antioxidant defenses in response to persistent salinity stress (Fig. 6A, Table S4).

The activities of antioxidant enzymes showed distinct variations influenced by genotype, salinity, and time. Catalase (CAT) activity exhibited stability under control conditions across various genotypes (32–38 U g⁻¹ FW), displayed minor decreases under S1 in Gi 171 and Sk 95, and experienced an increase under S2 in all genotypes, with the peak value noted in Gm 11 (48.18 U g⁻¹ FW). The activity of ascorbate peroxidase (APX) exhibited a notable increase in response to salinity stress, especially in Sk 95 under S1 at 17 days after sowing (DAS), with a measurement of 146.69 ± 0.01 U g⁻¹ FW. Meanwhile, Gm 11 and Mi 3 demonstrated significant rises under both S1 and S2, with values recorded at 24 DAS surpassing those at 17 DAS. The activity of glutathione reductase (GR) showed a notable increase under S1 in Gm 11 and Mi 3 at 17 DAS, measuring 34.31 ± 0.01 and 27.52 ± 0.01 U g⁻¹ FW, respectively. This was followed by a minor decrease under S2 in specific genotypes, yet the levels stayed elevated compared to the controls, which were approximately 17–18 U g⁻¹ FW.

The activity of polyphenol oxidase (PPO) showed an increase in response to varying levels of salinity and over time, achieving peak values in Gm 11 and Mi 3 under S2 at 24 days after sowing (9.42–9.45 µg g⁻¹ DW). In contrast, Sk 95 and Gi 171 demonstrated more moderate increases. In general, enzyme activities increased significantly at 24 DAS compared to 17 DAS across the different genotypes (Fig. 6B–E; Table S4).

## Multivariate analysis of physiological and biochemical responses

The principal component analysis for the morphological and biochemical parameters under different salinity concentrations (S1 and S2) at 17 DAS. PC1 and PC2 explain 45% and 20.7% of the total variation, respectively; PCA-biplot clearly demonstrates a strong separation between the control and salinity treatments along PC1, indicating that salinity stress is the primary source of variation among the measured traits. Control samples cluster tightly on the negative side of PC1, reflecting stable physiological and growth performance under non-stress conditions. In contrast, S1 and, especially, S2 treatments shift markedly toward the positive end of PC1, driven by higher levels of oxidative stress indicators (MDA, H₂O₂), secondary metabolites (TPC, TFC), and antioxidant enzymes (PPO, GR). The direction and length of the arrows show that these variables contribute strongly and positively to PC1 under high salinity.

**Fig. 7:**
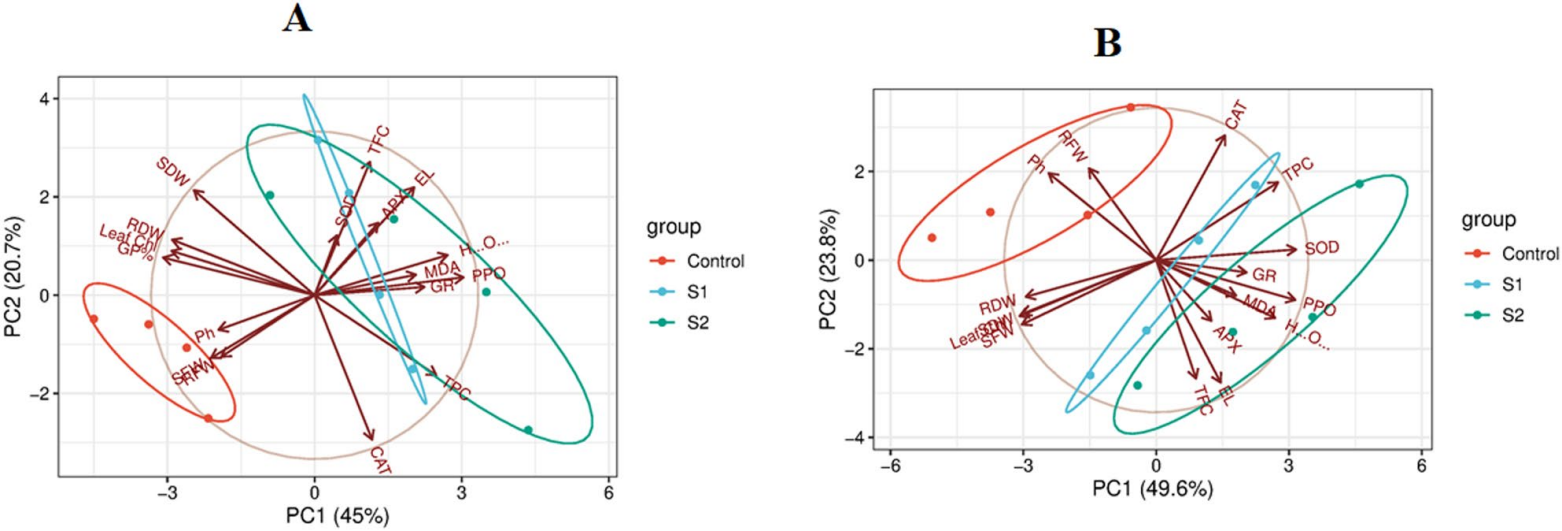
Principal component analysis (PCA) biplot illustrating the multivariate distribution of wheat genotypes under control and salinity treatments (S1 and S2). **A**: PCA for the genotypes at 17 DAS; **B**: PCA for genotypes at 24 DAS

Meanwhile, growth parameters such as SDW, RDW, SRW, leaf chlorophyll, and photosynthetic rate load negatively on PC1, indicating their decline as salinity increases. The separation between S1 and S2 suggests a progressive alteration in physiological behavior, with S2 showing the most significant divergence and the strongest association with stress-response traits. This pattern confirms that increased salinity triggers increased oxidative stress and defense activation while suppressing growth-related traits. Overall, the PCA pattern highlights a clear trade-off between growth and stress-response mechanisms, with salinity severity dictating the dominant physiological profile as shown in Fig. 7A.

Principal component analysis (PCA) was performed on morphological and biochemical characteristics of different wheat genotypes to evaluate clustering and relationships among the genotypes under varying levels of salt stress (S1 and S2) at 24 days after sowing. PC1 and PC2 explain 49.6 % and 23.8 % of the total variation, respectively. The information presented in Fig. 7B reveals that PCA1 accounted for 49.6% of the variation, whereas PCA2 accounted for 23.8%. Using principal component analysis (PCA), distinct differentiation among wheat genotypes was observed. In particular, three groups were delineated, with the first group representing genotypes positioned in the upper quadrant of the figure, influenced by plant height and root fresh weight (RFW). The second group of genotypes within S1, positioned centrally in the quadrant of the figure, is influenced by the total phenolic content (TPC). The third group consisted of genotypes subjected to S2 treatment, located in the lower quadrant of the figure, and influenced by the antioxidant enzymes SOD, PPO, and APX, as well as total flavonoid content (TFC). The magnitude of the arrow signifies the strength of the variable, whereas the direction of the arrow denotes (Fig. 7B).

**Fig. 8:**
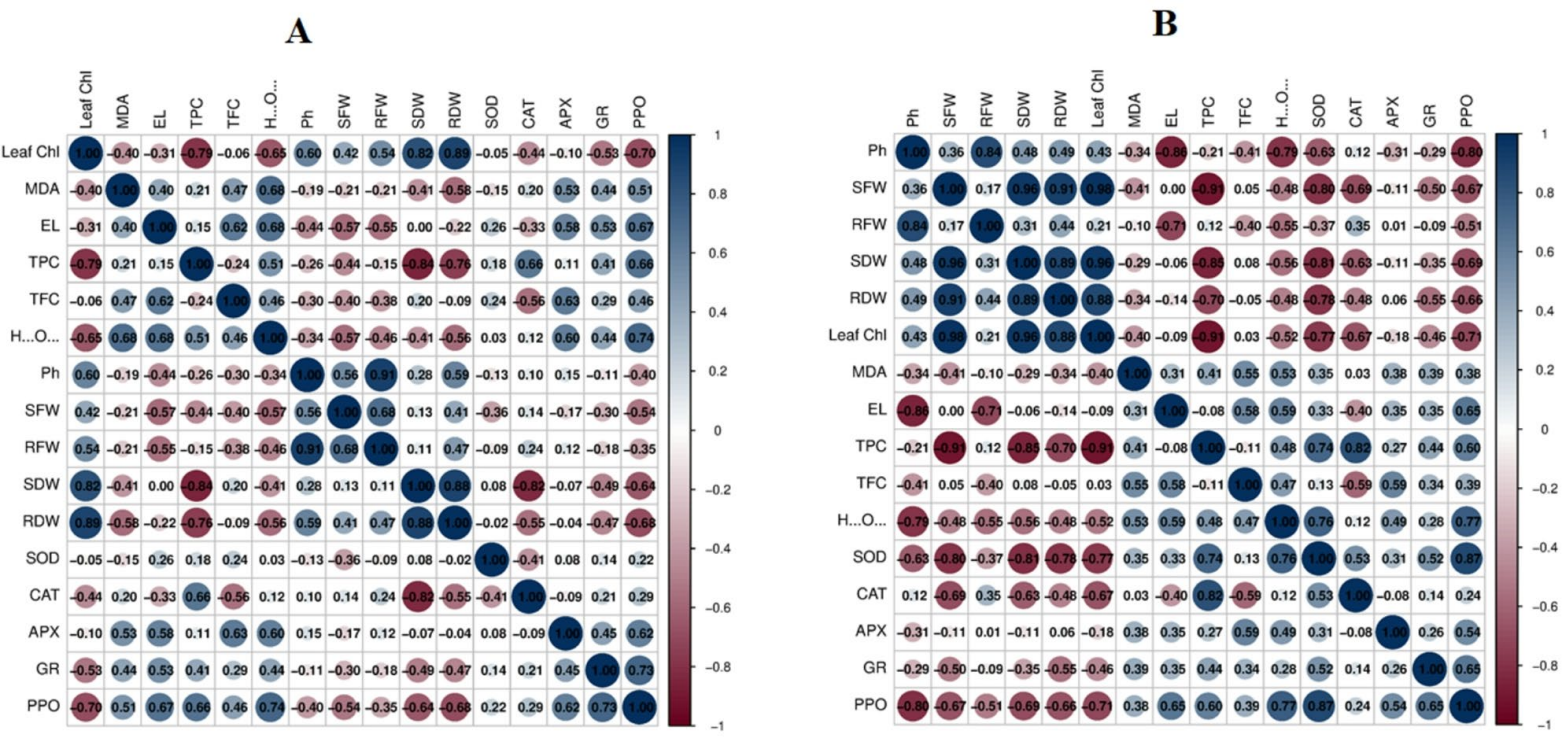
Pearson correlation correlogram based on the correlation coefficients of morphological, biochemical and antioxidant enzymes activities for the four genotypes treated with two concentrations of salinity (S1 & S2) **A**: Correlation at 17 DAS; **B**: Correlation at 24 DAS. The blue colour indicates the positive correlation between measurements, while the red colour assumes the negative one

The Pearson correlation coefficient between morphological, biochemical, and antioxidant enzyme activity for the wheat genotypes treated with S1 and S2 at 17 days after sowing (DAS) showed that the highest positive correlation was 0.91 between plant height and root fresh weight, followed by 0.89 between leaf chlorophyll and root dry weight. The lowest positive correlation was 0.03 between SOD and H_2_O_2_, followed by 0.08 between SOD and APX, and also by 0.11 between TPC and APX. The highest negative correlation was − 0.84 between TPC and SDW, followed by -0.82 between SDW and CAT. The lowest negative correlation was − 0.02 between RDW and SOD, followed by -0.04 between RDW and APX (Fig. 8A).

Regarding the Pearson correlation coefficients for morphological, biochemical, and antioxidant enzyme activity in wheat genotypes treated with S1 and S2 at 24 days after sowing (DAS), the highest positive correlation was 0.98 between SFW and leaf chlorophyll, followed by 0.96 between leaf chlorophyll and SDW and between SDW and SFW. The lowest positive correlation was 0.0 between El and SFW, followed by 0.03 between TFC and leaf chlorophyll, and also between CAT and MDA. The highest negative correlation was − 0.91 between TPC and leaf chlorophyll, and between TPC and SFW, followed by -0.86 between El and Plant height. The lowest negative correlation was − 0.05 between TFC and RDW, followed by -0.06 between El and SDW, and − 0.08 between El and TPC (Fig. 8B).

### Transcriptional responses of WRKYs, TaSOS1, and LEA-1genes

**Fig. 9:**
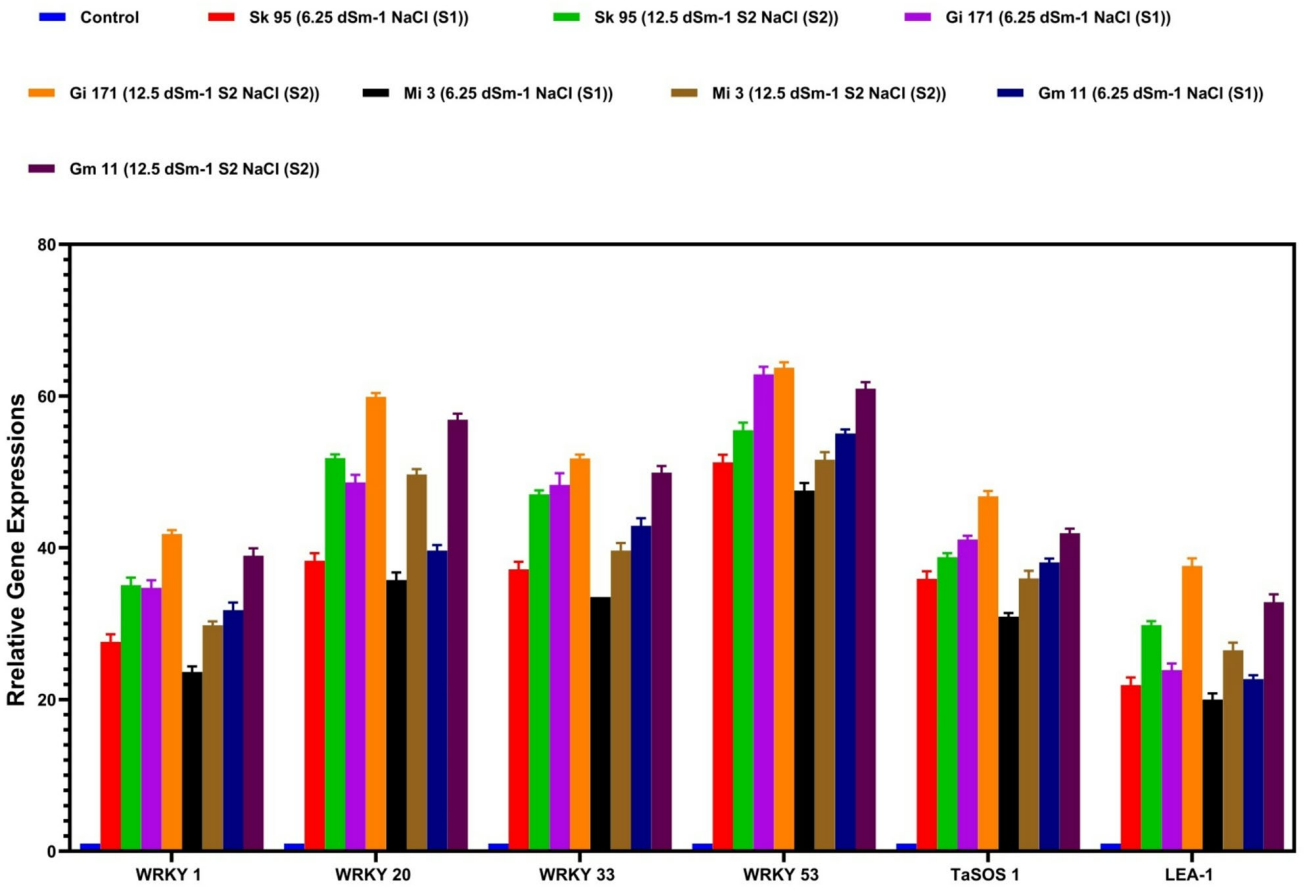
Relative expression levels of salinity-responsive genes (WRKY1, WRKY20, WRKY33, WRKY53, TaSOS1, and *LEA-1*) in four wheat genotypes (Sk 95, Gi 171, Mi 3, and Gm 11) under control and salt-stress conditions. Salt treatments included two NaCl concentrations: 6.25 dSm⁻¹ (S1) and 12.5 dSm⁻¹ (S2). Expression levels are presented as percentages relative to the control. Data are means ± standard error (*n* = 3)

**Fig. 10:**
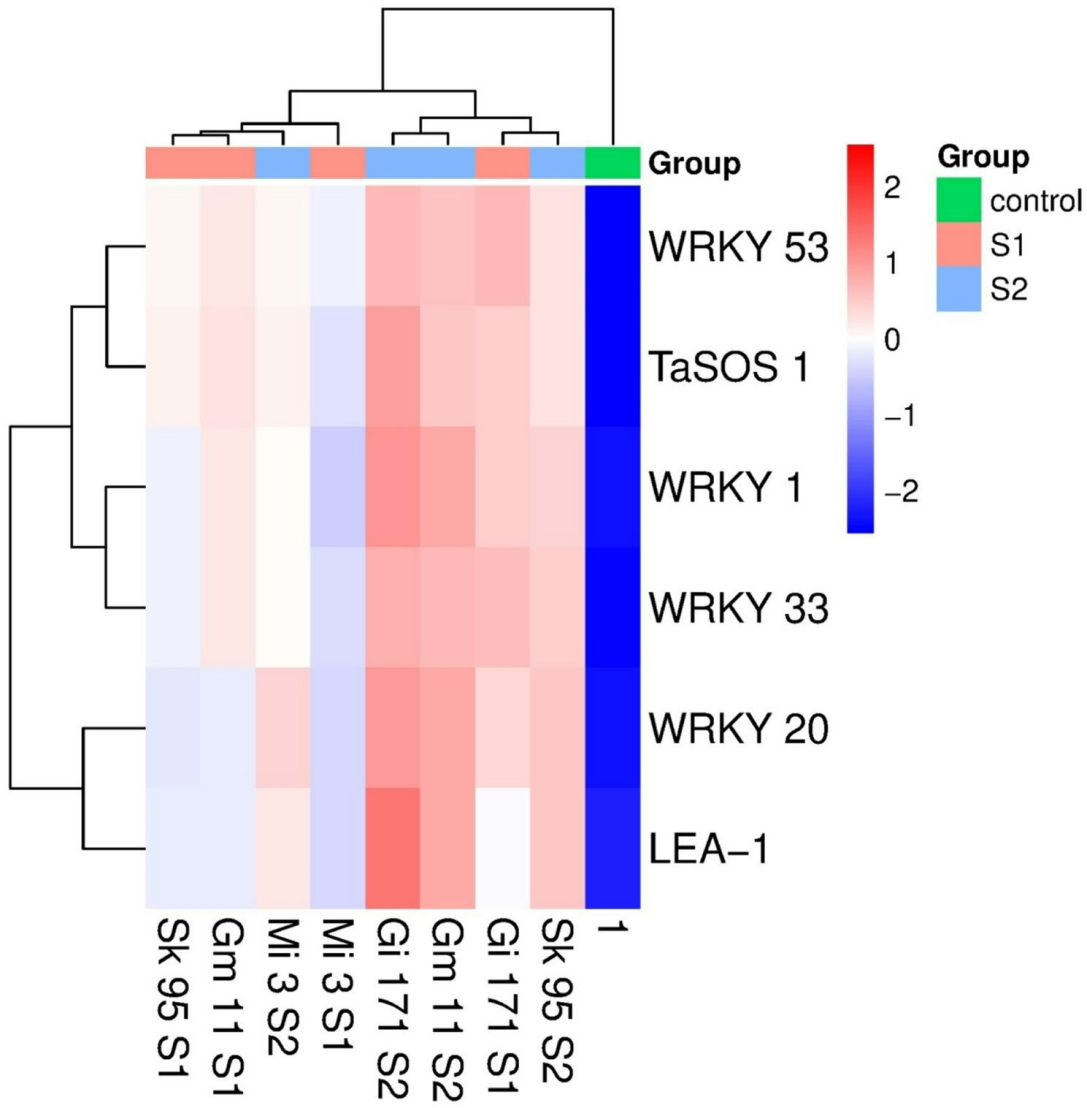
Hierarchical clustering heatmap of relative expression levels of LEA-1, TaSOS1 genes, and WRKY1, WRKY20, WRKY33 and, WRKY53 transcripts in four different *T.* *aestivum* genotypes grown for10 and 20 days under three salinity levels S1 and S2 (6.25 dSm^− 1^, and 12.5 dSm^− 1^ NaCl) and control

Salinity stress induced significant upregulation of WRKY1, WRKY20, WRKY33, WRKY53, TaSOS1, and LEA-1 in all genotypes (Figs. 9 and 10).The magnitude of gene induction increased with salinity level and was generally higher in Sakha 95 and Giza 171 than in Misr 3 and Gemmiza 11. WRKY20 and WRKY33 showed the strongest transcriptional responses under severe salinity, while TaSOS1 and LEA-1 were consistently upregulated across genotypes. Hierarchical clustering revealed precise genotype- and treatment-dependent expression patterns. Saline stress markedly enhanced the expression of all analyzed genes (WRKY1, WRKY20, WRKY33, WRKY53, TaSOS1, and LEA-1) across the four wheat genotypes, with induction typically increasing with increasing saline concentration (Fig. 9). WRKY1 expression was significantly elevated in Sakha 95 and Giza 171, reaching 34.08 and 41.39, respectively, under 12.5 dSm⁻¹ NaCl, whereas Misr 3 and Gemmiza 11 exhibited relatively lower induction. WRKY20 and WRKY33 showed the most pronounced transcriptional responses to extreme salinity, especially in Giza 171 and Gemmiza 11, where transcript levels were 50-fold higher than in the control.

WRKY53 exhibited a genotype-dependent expression pattern, with Giza 171 demonstrating the highest basal expression and only moderate increases under saline conditions, while the other genotypes showed lesser, yet substantial, induction. TaSOS1 transcripts were markedly raised across all genotypes, with the most significant expression recorded in Giza 171 (46.57) under high salinity, whereas Sakha 95, Misr 3, and Gemmiza 11 displayed moderate increases. LEA-1 expression was markedly elevated by salt, with the most pronounced upregulation observed in Giza 171 and Gemmiza 11 at 12.5 dSm⁻¹ NaCl, while Sakha 95 and Misr 3 exhibited lower, nevertheless significant transcript accumulation. The hierarchical clustering heatmap illustrates distinct genotype- and treatment-dependent expression patterns of WRKY1, WRKY20, WRKY33, WRKY53, TaSOS1, and LEA-1 (Fig. 10). The intensity of red color indicates greater relative transcript abundance, while blue indicates diminished expression levels. Samples subjected to salinity treatment, especially under S2, exhibited intense red hues across most genes, whereas control samples were primarily characterized by blue to neutral tones. Giza 171 and Sakha 95 showed more pronounced red signals under salt stress, indicating greater gene induction, whereas Misr 3 and Gemmiza 11 exhibited reduced color intensity.

## Discussion

Salinity stress severely constrains wheat growth by disrupting water relations, ionic balance, and cellular redox homeostasis [77, 78]. In this study, apparent genotypic variation among the four wheat cultivars enabled distinction between salt-tolerant (Sakha 95 and Giza 171) and salt-sensitive (Misr 3 and Gemmiza 11) responses at morphological, biochemical, and transcriptional levels.

Reductions in germination percentage, plant height, and biomass at increasing NaCl concentrations are consistent with previous reports of salinity-induced growth inhibition via osmotic stress and ion toxicity [79, 80]. However, tolerant genotypes maintained significantly higher growth parameters across both sampling times, indicating superior physiological adjustment. The more substantial decline observed at 24 DAS compared with 17 DAS suggests cumulative stress effects during prolonged salinity exposure (Sudhir & Murthy, 2004; El-Hendawy et al., 2017). The findings indicate that Sakha 95 and Giza 171 exhibit enhanced salinity tolerance, sustaining germination and seedling growth in adverse conditions. In contrast, Misr 3 and Gemmiza 11 show greater sensitivity, with significant growth inhibition at higher salinity levels. The differential responses highlight the importance of genotype selection in breeding programs to enhance wheat salt tolerance. Root biomass displayed comparable trends, although the reductions were typically less pronounced than those observed in shoots, indicating a preferential allocation of resources to roots in saline conditions.

The temporal increase from 17 to 24 days after sowing indicates persistent membrane peroxidation during extended stress conditions. The observations support the significant role of oxidative stress in facilitating salinity-induced cellular damage in wheat seedlings. EL values increased consistently over time, indicating a gradual decline in membrane stability as salinity stress persisted from 17 to 24 DAS. The results highlight the sensitivity of wheat genotypes to ionic imbalance and osmotic stress at the membrane level. The elevation at 24 DAS was consistently higher than at 17 DAS, indicating cumulative oxidative stress over time. The findings of H_2_O_2_ support the idea that salinity stress triggers oxidative bursts, requiring the simultaneous activation of antioxidative pathways, including the synthesis of phenolics and flavonoids. Increased levels of malondialdehyde, hydrogen peroxide, and electrolyte leakage under salinity confirm that oxidative stress is a significant component of salinity-induced injury [81, 82]. Sensitive genotypes exhibited more serious oxidative damage, whereas tolerant genotypes maintained lower levels despite equivalent stress, suggesting more effective redox buffering rather than reduced stress perception. Similar genotype-dependent patterns have been reported in wheat under salinity stress [81, 83–87].

Antioxidant enzymes responded in a coordinated manner rather than as isolated components. In tolerant genotypes, concurrent induction of SOD, APX, CAT, GR, and PPO supports efficient sequential detoxification of reactive oxygen species [88, 89]. In contrast, sensitive genotypes displayed less balanced enzyme responses accompanied by more serious oxidative damage, indicating that uncoordinated antioxidant activation is insufficient for effective stress mitigation [90, 91].

Non-enzymatic antioxidants also contributed to salinity responses, as reflected by increased phenolic and flavonoid accumulation. These compounds are known to participate in redox buffering and membrane protection, particularly under prolonged stress [81, 92]. However, their accumulation alone did not prevent oxidative damage in sensitive genotypes, emphasizing the importance of integrated antioxidant regulation.

Salinity stress induced significant upregulation of WRKY1, WRKY20, WRKY33, and WRKY53 across all genotypes, indicating their involvement in salinity-responsive transcriptional programs [81, 93, 94]. Stronger induction in tolerant genotypes suggests an association with enhanced stress responsiveness. However, in the absence of functional validation, these data do not establish a causal regulatory role for WRKY genes, which should therefore be regarded as transcriptional indicators and potential contributors rather than definitive master regulators [95, 96].

The concurrent upregulation of WRKY genes with TaSOS1 and LEA-1 suggests coordinated activation of transcriptional programs related to ion homeostasis and cellular protection. Elevated TaSOS1 expression may reflect enhanced Na⁺ management capacity [97], while increased LEA-1 expression likely contributes to macromolecular stabilization under osmotic stress [98, 99]. Although regulatory relationships cannot be inferred from expression data alone, the synchronized patterns support the involvement of integrated stress-responsive networks.

Temporal differences between 17 and 24 DAS indicate that salinity responses are dynamic. Early responses involved rapid transcriptional and antioxidant activation, whereas prolonged exposure led to increased oxidative pressure and sustained defense activity. Tolerant genotypes maintained more stable responses over time, while sensitive genotypes exhibited progressive physiological decline, consistent with earlier reports of stress acclimation dynamics [100, 101].

Multivariate analyses further highlighted trade-offs between growth-related traits and stress-response parameters, reinforcing the integrative nature of salinity tolerance. A limitation of this study is the lack of functional genetic validation, which restricts causal inference. Future studies employing transgenic or gene-editing approaches will be necessary to confirm regulatory relationships. Nevertheless, the integrated dataset presented here provides a solid framework for identifying candidate traits and genes associated with salinity tolerance in wheat.

Principal component analysis (PCA) enabled an integrative assessment of the relationships among growth, oxidative stress, and antioxidant traits under salinity stress. It clarified the variables most strongly contributing to genotype separation. The first principal component (PC1), which accounted for the most significant proportion of total variance, was primarily driven by oxidative stress indicators (malondialdehyde, hydrogen peroxide, and electrolyte leakage) together with antioxidant-related traits (SOD, PPO, total phenolics, and total flavonoids), indicating that redox imbalance and defense activation were the dominant sources of variation under salinity stress [87, 96]. In contrast, growth-related parameters and chlorophyll content loaded negatively on PC1, reflecting their consistent decline with increasing salinity [102]. This opposing loading pattern highlights a trade-off between growth maintenance and stress defense. Salt-tolerant genotypes clustered in regions characterized by relatively preserved growth traits and moderate antioxidant activation, whereas sensitive genotypes clustered with high oxidative stress markers and reduced growth performance. Similar PCA-based differentiation between tolerant and sensitive genotypes has been reported in wheat and other crops under salinity stress [81, 96], supporting the interpretation that tolerance is associated with balanced physiological and biochemical adjustment rather than extreme stress-response activation. Heatmaps achieve their most excellent effectiveness when combined with hierarchical clustering that accounts for distance or similarity measures. One approach to examine the relationship between plant characteristics and their ability to withstand salinity is to use principal component analysis, a method that simultaneously evaluates multiple variables.PCA elucidates the most significant variations in interaction through morphological and physiological characteristics [103].

The present study was conducted under short-term, pot-based greenhouse conditions, which represent a controlled environment for dissecting early salinity responses but do not fully capture the complexity of field conditions. Root confinement, uniform soil composition, and relatively brief exposure duration may influence the magnitude and dynamics of physiological and molecular responses. In addition, seedling-stage assessments may not fully reflect responses at later developmental stages or under chronic salinity stress encountered in agricultural systems. Therefore, while the observed genotype-specific differences provide valuable insight into early salinity response mechanisms, extrapolation to field performance should be made with caution. Future studies integrating long-term field trials, multiple developmental stages, and functional genetic validation will be essential to confirm the relevance of these findings for breeding and crop improvement programs.

## Conclusion

The impact of salinity stress notably hindered wheat growth and physiological performance, with effects varying by genotype. Sakha 95 and Giza 171 demonstrated greater resilience than Gemmiza 11 and Misr 3, as indicated by improved growth, greater chlorophyll retention, and lower levels of membrane damage under saline conditions. Improved tolerance was associated with the coordinated activation of protective mechanisms, including SOD, CAT, APX, GR, and PPO, along with heightened phenolic accumulation, suggesting a holistic redox-regulatory response rather than separate enzymatic actions. Salinity elicited distinct transcriptional responses by genotype, with more pronounced upregulation of WRKY1, WRKY20, WRKY33, WRKY53, TaSOS1, and LEA-1 in tolerant genotypes. The observed expression patterns suggest a potential role for these genes in stress-responsive regulatory networks; however, their specific functions require further validation. Multivariate analyses indicated a strong association between reduced oxidative stress and the clustering of traits related to tolerance in saline conditions. The identification of tolerant genotypes, along with their physiological and molecular markers, offers valuable opportunities to enhance wheat breeding initiatives aimed at optimizing performance in salt-impacted agricultural environments. 

## Supplementary Information

Supplementary Material 1

## Data Availability

The relevant datasets supporting the results of this article are included within the article. There is no sequencing data generated in the current study.
