# Supplementary material for: Transcriptional signatures of salinity tolerance in Egyptian wheat: unveiling WRKY-mediated defense mechanisms
Source: BMC Plant Biol. 2026 Mar 23;26:640. doi: 10.1186/s12870-026-08483-0 (PMC13063623; doi:10.1186/s12870-026-08483-0)
Supplement: Supplementary file 1 — Supplementary Material 1 [file 12870_2026_8483_MOESM1_ESM.docx]

Table S1. Environmental conditions (day and night temperature, mean daily temperature, and relative humidity) during different experimental periods and growth stages of wheat plants from germination to stress treatment (September–October 2025).

| **Experimental Period** | **Growth Stage** | **Day Temperature (°C)** | **Night Temperature (°C)** | **Mean Daily Temperature (°C)** | **Relative Humidity (%)** |
| --- | --- | --- | --- | --- | --- |
| Early September 2025 | Germination | 18–22 | 9–13 | 15.5–17.5 | 60–70 |
| Mid -September 2025 | Seedling establishment | 20–23 | 11–15 | 17.0–19.0 | 60–70 |
| Late September 2025 | Early vegetative growth | 22–25 | 14–17 | 18.5–21.0 | 60–70 |
| Early October 2025 | Vegetative growth | 21–24 | 15–18 | 18.0–21.0 | 60–70 |
| Mid–Late October 2025 | Stress treatment period | 20–23 | 16–19 | 18.0–21.0 | 60–70 |

| Treatment | Giza 171 C | Giza 171 S1 | Giza 171 S2 | Gemmiza 11 C | Gemmiza 11 S1 | Gemmiza 11 S2 | Sakha 95 C | Sakha 95 S1 | Sakha 95 S2 | Misr 3 C | Misr 3 S1 | Misr 3 S2 |
| --- | --- | --- | --- | --- | --- | --- | --- | --- | --- | --- | --- | --- |
| GP% | 100 | 95.3 | 92.0 | 100 | 86.3 | 81.3 | 100 | 97.7 | 94.7 | 100 | 90.0 | 86.0 |
| Ph 17 DAS (cm) | 16.30 ±0.01 | 15.66 ±0.02 | 14.31 ±0.01 | 18.62 ±0.01 | 17.84 ±0.01 | 15.05 ±0.01 | 19.20 ±0.01 | 17.97 ±0.01 | 14.67 ±0.01 | 15.74 ±0.01 | 14.90 ±0.01 | 13.28 ±0.01 |
| Ph 24 DAS (cm) | 30.80 ±0.01 | 28.12 ±0.01 | 26.33 ±0.01 | 32.40 ±0.01 | 28.61 ±0.01 | 26.34 ±0.01 | 31.62 ±0.01 | 27.83 ±0.01 | 25.57 ±0.01 | 29.08 ±0.01 | 26.30 ±0.01 | 21.41 ±0.01 |
| SFW 17 DAS (g) | 3.08 ±0.01 | 2.82 ±0.01 | 2.57 ±0.01 | 3.25 ±0.01 | 3.09 ±0.01 | 2.91 ±0.01 | 3.17 ±0.01 | 2.96 ±0.01 | 2.84 ±0.01 | 3.85 ±0.01 | 2.52 ±0.01 | 2.37 ±0.01 |
| SFW 24 DAS (g) | 3.65 ±0.01 | 3.23 ±0.01 | 2.97 ±0.01 | 2.84 ±0.01 | 2.71 ±0.01 | 2.50 ±0.01 | 3.90 ±0.01 | 3.71 ±0.01 | 3.55 ±0.01 | 3.17 ±0.01 | 3.06 ±0.01 | 2.81 ±0.01 |
| RFW 17 DAS (g) | 1.30 ±0.01 | 1.25 ±0.01 | 1.17 ±0.01 | 1.50 ±0.01 | 1.41 ±0.01 | 1.27 ±0.01 | 1.42 ±0.01 | 1.37 ±0.01 | 1.30 ±0.01 | 1.34 ±0.01 | 1.23 ±0.01 | 1.18 ±0.01 |
| RFW 24 DAS (g) | 1.70 ±0.01 | 1.62 ±0.01 | 1.53 ±0.01 | 1.81 ±0.01 | 1.75 ±0.01 | 1.65 ±0.01 | 1.75 ±0.01 | 1.66 ±0.01 | 1.61 ±0.01 | 1.59 ±0.01 | 1.55 ±0.01 | 1.49 ±0.01 |
| SDW 17 DAS (g) | 1.18 ±0.01 | 1.12 ±0.01 | 1.06 ±0.01 | 0.98 ±0.01 | 0.87 ±0.01 | 0.76 ±0.01 | 1.25 ±0.01 | 1.20 ±0.01 | 1.15 ±0.01 | 1.06 ±0.01 | 1.02 ±0.01 | 0.96 ±0.01 |
| SDW 24 DAS (g) | 1.33 ±0.01 | 1.29 ±0.01 | 1.17 ±0.01 | 1.14 ±0.01 | 1.16 ±0.01 | 1.09 ±0.01 | 1.41 ±0.01 | 1.36 ±0.01 | 1.29 ±0.01 | 1.24 ±0.01 | 1.15 ±0.01 | 1.09 ±0.01 |
| RDW 17 DAS (g) | 0.70 ±0.01 | 0.60 ±0.01 | 0.54 ±0.01 | 0.61 ±0.01 | 0.55 ±0.01 | 0.47 ±0.01 | 0.72 ±0.01 | 0.70 ±0.01 | 0.64 ±0.01 | 0.63 ±0.01 | 0.61 ±0.01 | 0.50 ±0.01 |
| RDW 24 DAS (g) | 0.76 ±0.01 | 0.65 ±0.01 | 0.60 ±0.01 | 0.70 ±0.01 | 0.63 ±0.01 | 0.55 ±0.01 | 0.89 ±0.01 | 0.84 ±0.01 | 0.81 ±0.01 | 0.70 ±0.01 | 0.60 ±0.01 | 0.60 ±0.01 |

Table S2. Effect of different salinity concentrations (dSm⁻¹) on four *T. aestivum* genotypes seedling growth parameters (plant height, shoot fresh weight, root fresh weight, shoot dry weight, and root dry weight) for 10 and 20 days subjected to salinity stress (17, 24 DAS). Mean ± SD represents the mean of five replicates and standard deviation.

| Parameter | Unit | Giza (Gi 171) C | Giza (Gi 171) S1 | Giza (Gi 171) S2 | Gemmiza (Gm 11) C | Gemmiza (Gm 11) S1 | Gemmiza (Gm 11) S2 | Sakha (Sk 95) C | Sakha (Sk 95) S1 | Sakha (Sk 95) S2 | Misr (Mi 3) C | Misr (Mi 3) S1 | Misr (Mi 3) S2 |
| --- | --- | --- | --- | --- | --- | --- | --- | --- | --- | --- | --- | --- | --- |
| Leaf Chl 17 DAS | mg/g FW | 3.11 ± 0.006 | 2.92 ± 0.01 | 2.80 ± 0.01 | 2.98 ± 0.01 | 2.85 ± 0.01 | 2.65 ± 0.006 | 3.31 ± 0.01 | 3.177 ± 0.006 | 3.05 ± 0.01 | 2.94 ± 0.01 | 2.77 ± 0.01 | 2.70 ± 0.01 |
| Leaf Chl 24 DAS | mg/g FW | 2.73 ± 0.01 | 2.60 ± 0.01 | 2.52 ± 0.01 | 2.44 ± 0.01 | 2.38 ± 0.01 | 2.30 ± 0.01 | 2.87 ± 0.01 | 2.76 ± 0.01 | 2.61 ± 0.01 | 2.53 ± 0.006 | 2.50 ± 0.01 | 2.40 ± 0.01 |
| MDA 17 DAS | nmol/g FW | 37.41 ± 0.006 | 68.11 ± 0.01 | 71.93 ± 0.01 | 39.81 ± 0.01 | 74.15 ± 0.01 | 81.51 ± 0.01 | 34.20 ± 0.01 | 48.16 ± 0.01 | 69.20 ± 0.01 | 40.61 ± 0.01 | 8.12 ± 0.01 | 71.92 ± 0.01 |
| MDA 24 DAS | nmol/g FW | 46.16 ± 0.01 | 72.65 ± 0.01 | 75.55 ± 0.01 | 49.70 ± 0.01 | 68.12 ± 0.01 | 74.50 ± 0.01 | 32.64 ± 0.02 | 46.13 ± 0.01 | 64.51 ± 0.01 | 38.16 ± 0.01 | 7.98 ± 0.01 | 68.12 ± 0.01 |
| EL 17 DAS | % | 37.74 ± 0.006 | 74.51 ± 0.01 | 57.11 ± 0.01 | 23.90 ± 0.01 | 57.93 ± 0.01 | 63.11 ± 0.02 | 47.05 ± 0.02 | 67.10 ± 0.03 | 86.32 ± 0.02 | 47.52 ± 0.02 | 69.20 ± 0.01 | 78.21 ± 0.01 |
| EL 24 DAS | % | 39.44 ± 0.01 | 75.18 ± 0.01 | 61.49 ± 0.01 | 24.12 ± 0.01 | 58.07 ± 0.01 | 63.42 ± 0.01 | 51.62 ± 0.02 | 69.81 ± 0.01 | 87.14 ± 0.01 | 52.42 ± 0.01 | 65.91 ± 0.01 | 88.12 ± 0.01 |
| TPC 17 DAS | µmol/g GAE | 56.18 ± 0.01 | 57.52 ± 0.02 | 63.33 ± 0.01 | 73.41 ± 0.01 | 75.16 ± 0.01 | 79.52 ± 0.01 | 55.41 ± 0.01 | 57.12 ± 0.01 | 61.15 ± 0.01 | 56.92 ± 0.01 | 77.19 ± 0.01 | 81.19 ± 0.01 |
| TPC 24 DAS | µmol/g GAE | 59.19 ± 0.01 | 61.52 ± 0.02 | 65.78 ± 0.01 | 74.65 ± 0.01 | 77.13 ± 0.01 | 81.64 ± 0.01 | 56.30 ± 0.01 | 58.43 ± 0.01 | 64.83 ± 0.01 | 62.41 ± 0.01 | 66.51 ± 0.01 | 69.03 ± 0.01 |
| TFC 17 DAS | µmol/g QE | 2.52 ± 0.01 | 6.71 ± 0.01 | 6.93 ± 0.01 | 3.09 ± 0.01 | 3.76 ± 0.01 | 3.90 ± 0.02 | 2.48 ± 0.02 | 6.53 ± 0.01 | 6.81 ± 0.01 | 3.15 ± 0.01 | 3.64 ± 0.01 | 3.71 ± 0.01 |
| TFC 24 DAS | µmol/g QE | 2.61 ± 0.01 | 6.74 ± 0.01 | 7.13 ± 0.01 | 3.22 ± 0.01 | 3.61 ± 0.01 | 4.13 ± 0.01 | 2.55 ± 0.01 | 6.17 ± 0.01 | 6.56 ± 0.01 | 2.85 ± 0.01 | 3.36 ± 0.01 | 3.91 ± 0.01 |
| H₂O₂ 17 DAS | µmol/g FW | 14.91 ± 0.01 | 35.61 ± 0.01 | 60.81 ± 0.01 | 19.30 ± 0.01 | 40.52 ± 0.01 | 65.71 ± 0.10 | 15.62 ± 0.02 | 17.41 ± 0.01 | 71.72 ± 0.01 | 16.52 ± 0.01 | 37.81 ± 0.01 | 62.43 ± 0.01 |
| H₂O₂ 24 DAS | µmol/g FW | 18.30 ± 0.01 | 19.61 ± 0.01 | 71.82 ± 0.01 | 20.51 ± 0.01 | 43.61 ± 0.01 | 71.90 ± 0.02 | 17.30 ± 0.02 | 44.50 ± 0.01 | 58.31 ± 0.01 | 19.31 ± 0.01 | 39.42 ± 0.01 | 66.50 ± 0.01 |

Table 3. Effect of different salinity concentrations (dSm⁻¹) on leaf chlorophyll content (Leaf Chl), malondialdehyde (MDA), electrolyte leakage (EL), total phenolic content (TPC), total flavonoid content (TFC), and hydrogen peroxide (H₂O₂) in four *T.* *aestivum* genotypes at 17 and 24 DAS. Values are expressed as mean ± SD of five replicates.

Table 4. Effect of different salinity concentrations (dSm⁻¹) on antioxidant enzyme activities (SOD, CAT, APX, GR, and PPO) of four *T. aestivum* genotypes at 17 and 24 DAS. Values represent mean ± SD of five replicates.

| Genotype | Treatment | SOD (17 DAS) | SOD (24 DAS) | CAT (17 DAS) | CAT (24 DAS) | APX (17 DAS) | APX (24 DAS) | GR (17 DAS) | GR (24 DAS) | PPO (17 DAS) | PPO (24 DAS) |
| --- | --- | --- | --- | --- | --- | --- | --- | --- | --- | --- | --- |
| Giza (Gi 171) | C | 49.81 ± 0.01 | 52.41 ± 0.01 | 33.11 ± 0.01 | 33.81 ± 0.01 | 114.23 ± 0.01 | 115.22 ± 0.01 | 17.62 ± 0.01 | 18.11 ± 0.01 | 3.15 ± 0.01 | 3.16 ± 0.01 |
| Giza (Gi 171) | S1 | 62.63 ± 0.01 | 72.51 ± 0.01 | 27.29 ± 0.01 | 28.11 ± 0.01 | 126.91 ± 0.01 | 123.41 ± 0.01 | 33.54 ± 0.01 | 32.21 ± 0.01 | 6.81 ± 0.01 | 6.91 ± 0.01 |
| Giza (Gi 171) | S2 | 89.51 ± 0.01 | 91.41 ± 0.01 | 28.91 ± 0.01 | 28.74 ± 0.01 | 119.41 ± 0.01 | 125.61 ± 0.01 | 20.91 ± 0.01 | 24.51 ± 0.01 | 6.51 ± 0.01 | 7.11 ± 0.01 |
| Gemmiza (Gm 11) | C | 74.12 ± 0.01 | 75.62 ± 0.01 | 38.18 ± 0.01 | 41.31 ± 0.01 | 122.53 ± 0.01 | 131.41 ± 0.01 | 18.31 ± 0.01 | 18.91 ± 0.01 | 5.19 ± 0.01 | 5.32 ± 0.01 |
| Gemmiza (Gm 11) | S1 | 111.21 ± 0.01 | 89.21 ± 0.01 | 39.51 ± 0.01 | 40.42 ± 0.01 | 127.31 ± 0.01 | 128.91 ± 0.01 | 34.31 ± 0.01 | 35.71 ± 0.01 | 8.12 ± 0.01 | 8.23 ± 0.01 |
| Gemmiza (Gm 11) | S2 | 12.07 ± 0.01 | 123.71 ± 0.01 | 47.18 ± 0.01 | 48.17 ± 0.01 | 127.52 ± 0.01 | 130.32 ± 0.01 | 28.61 ± 0.01 | 27.32 ± 0.01 | 9.21 ± 0.01 | 9.42 ± 0.01 |
| Sakha (Sk 95) | C | 56.71 ± 0.01 | 57.34 ± 0.01 | 32.60 ± 0.01 | 32.91 ± 0.01 | 111.72 ± 0.01 | 111.91 ± 0.01 | 17.55 ± 0.01 | 17.61 ± 0.01 | 3.19 ± 0.01 | 3.26 ± 0.01 |
| Sakha (Sk 95) | S1 | 78.31 ± 0.01 | 79.45 ± 0.01 | 27.30 ± 0.01 | 28.41 ± 0.01 | 146.69 ± 0.01 | 147.21 ± 0.01 | 24.41 ± 0.01 | 24.81 ± 0.01 | 7.61 ± 0.01 | 7.60 ± 0.01 |
| Sakha (Sk 95) | S2 | 97.18 ± 0.01 | 62.71 ± 0.01 | 28.91 ± 0.01 | 28.65 ± 0.01 | 125.68 ± 0.01 | 137.65 ± 0.01 | 19.81 ± 0.01 | 20.11 ± 0.01 | 6.92 ± 0.01 | 7.14 ± 0.01 |
| Misr (Mi 3) | C | 62.31 ± 0.01 | 62.71 ± 0.01 | 32.08 ± 0.01 | 35.41 ± 0.01 | 113.62 ± 0.01 | 115.66 ± 0.01 | 18.91 ± 0.01 | 18.68 ± 0.01 | 4.31 ± 0.01 | 5.31 ± 0.01 |
| Misr (Mi 3) | S1 | 105.21 ± 0.01 | 93.54 ± 0.01 | 32.61 ± 0.01 | 35.92 ± 0.01 | 117.91 ± 0.01 | 119.64 ± 0.01 | 27.52 ± 0.01 | 26.39 ± 0.01 | 7.91 ± 0.01 | 8.26 ± 0.01 |
| Misr (Mi 3) | S2 | 98.11 ± 0.01 | 106.91 ± 0.01 | 33.64 ± 0.01 | 34.71 ± 0.01 | 119.42 ± 0.01 | 127.41 ± 0.01 | 22.81 ± 0.01 | 23.31 ± 0.01 | 7.31 ± 0.01 | 9.44 ± 0.01 |
